# *Morganella morganii* bloodstream infection affects the elderly in close contact with health care

**DOI:** 10.1016/j.ijregi.2024.100480

**Published:** 2024-10-24

**Authors:** Olof Karlbom, Torgny Sunnerhagen, Oskar Ljungquist

**Affiliations:** 1 Division of Infection Medicine, Department of Clinical Sciences, Lund University, Lund, Sweden; 2 Clinical Microbiology, Infection Prevention and Control, Office for Medical Services, Region Skåne, Lund, Sweden; 3 Department of Infectious Diseases, Helsingborg hospital, Helsingborg, Sweden

**Keywords:** Bacteremia, Bacterial infections, Antimicrobial resistance, Sepsis, Risk factors

## Abstract

**Objectives:**

We aimed to fill the gaps of knowledge concerning incidence rates and temporal trend, clinical features and risk factors for death for *Morganella morganii* bloodstream infections (BSIs) in south Sweden.

**Methods:**

This was an observational, population-based study including all patients with at least one blood culture positive for *M. morganii* between 2013 and 2023 in Skåne, south Sweden.

**Results:**

A total of 211 episodes of *M. morganii* BSI in 201 patients were registered during the study period. A urinary tract–focused BSI was associated with a reduced risk of 90-day mortality (*P* = 0.0029), whereas a primary BSI was associated with 90-day mortality (*P* = 0.0112). A multivariate analysis including age, gender, Charlson comorbidity index (CCI), and immunosuppression revealed that higher CCI (odds ratio [OR] 1.25, 95% confidence interval [CI] 1.07-1.47, *P =* 0.0059) and immunosuppression (OR 3.26, 95% CI 1.45-7.47, *P* = 0.0045) was independently associated with all-cause mortality.

**Conclusions:**

*M. morganii* BSI is a disease among the elderly that most often acquired in individuals with comorbidities that are in close contact with health care. A urinary tract focus was associated with reduced risk of 90-day mortality, and higher CCI and immunosuppression were associated with 90-day all-cause mortality in a multivariate analysis.

## Highlights

• In recent years there has been an increase in *morganii* bloodstream infections (BSIs).
• *Morganella morganii* BSIs affects the elderly.
• Polymicrobial growth was seen in 40% of episodes.
• Comorbidities and immunosuppression were associated with mortality.
• Only 22% of *M. morganii* BSI were community-acquired.

## Introduction

Bloodstream infections (BSIs) heavily burden health care and is a major cause of mortality worldwide [1]. A recent study reported that the overall incidence rate of BSIs in our setting was 307 per 100,000 person-years, with an average annual increase of 3% [2]. The increasing use of antimicrobials worldwide is driving the selection of resistant genes, and this is no exception for *Morganella morganii,* which can harbor genes expressing AmpC beta-lactamase, and other extended spectrum β-lactamases, which can confer resistance to several important antibiotics used in health care [3,4].

*M. morganii* is an enteric facultatively anaerobic gram-negative rod, first discovered by H. de R. Morgan in 1906 [5]. *M. morganii* is an opportunistic bacterium that can cause nosocomial, health care–associated, and community-acquired infections [6]. The severity of infections with *M. morganii* ranges from mild to life-threatening infections [6].

Previous population-based studies on incidence, clinical features, and outcome of *M. morganii* BSI are few. The annual crude incidence rate of *M. morganii* BSI has been estimated to 0.5 per 100,000 person-years in Canada and the overall age- and sex-standardized incidence rate was recently estimated to 0.92 per 100,000 person-years in Australia [7,8]. *M. morganii* has previously been referred to as an opportunistic bacterium, primarily associated with health care–related and nosocomial infections [7]. Health care facilities are high-risk environments for the spread of multidrug-resistant bacteria, which is why it is important to monitor the incidence of *M. morganii* BSI and the rate of resistance toward clinically important antimicrobials used in health care. Furthermore, the number of patients that are immunosuppressed due to medication have increased in recent years, which could potentially increase the risk of BSI in general [9]. To the best of our knowledge, few to no studies on *M. morganii* BSI originate from northern Europe. We wanted to fill the gaps of knowledge concerning the incidence rates and temporal trend, clinical features, and risk factors for mortality for *M. morganii* BSIs in this setting.

## Methods

### Setting and study type

This was an observational, population-based study including all patients with at least one blood culture positive for *M. morganii* between 2013 and 2023 in Skåne, south Sweden. Skåne had a population of almost 1.3 million people in 2013, which increased to 1.4 million inhabitants in 2023. The inclusion criterion was growth of *M. morganii* in blood cultures during the study period, and the exclusion criterion was inaccessibility to medical records and patients cultured postmortem. Data (personal identification numbers) of patients with growth of *M. morganii* in blood culture were retrieved from the department clinical microbiology, Region Skåne (LIMS-systems ww-lab, Autonik, Nyköping, Sweden). This is the only clinical microbiology laboratory in Skåne, serving public hospitals, the single private hospital in the region, and outpatient clinics. It is standard procedure to obtain four bottles from each patient when acquiring blood cultures.

During the study period, matrix-assisted laser desorption/ionization–time of flight mass spectrometry (Bruker Daltonics, using the Bruker MBT Compass library) was used as the standard method for species determination. For blood culture, the BacT/ALERT system (bioMérieux, Inc., Marcy-l’Étoile, France) was used in until December 2014, when it was replaced by the BACTEC FX system (BectonDickinson, Franklin Lakes, USA).

### Review of medical records and definitions

Medical records of patients with blood cultures positive for *M. morganii* were reviewed according to a predefined study protocol (detailed in Table S1). Medical records were accessed through Melior (Melior, Siemens Healthcare Service, Upplands Väsby, Sweden), the medical record system used in Skåne. The Charlson comorbidity index (CCI) was used to assess patient comorbidities [10]. Immunosuppression was defined as a patient with previous organ transplantation, or ongoing immunosuppressive medication, such as tumor necrosis factor-αinhibitor or corticosteroid treatment exceeding 15 mg prednisolone per day, or previous stem cell transplantation, or primary immune defect, or ongoing cancer-treatment such as chemotherapy, dialysis or severe chronic kidney disease, or ongoing treatment for autoimmune disease. Community-acquired, nosocomial, and health care–associated infection were defined according to a previously published definition [11]. Disease severity was assessed using National Early Warning Score (NEWS) [12]. The Sepsis-3 criteria were used to define septic shock [13]. Polymicrobial blood culture was defined as the presence of any pathogen other than *M. morgani* in at least one blood culture bottle. Primary BSI was defined as no other apparent source of *M. morganii* found during the chart review, which was done by a final year medical student. Only one episode of *M. morganii* per hospitalization was included in the study.

### Statistical methods

For the univariate analyses, the chi-square test was used for binary parameters, Mann–Whitney U-test for non-parametric parameters, or *t*-test for parametric parameters. For the multivariate analyses, we used multiple logistic regression, expressed with odds ratios (ORs) and confidence intervals (CIs). To reduce the risk of overfitting, the number of variables included in the model was restricted to four due to the low number of events. All analyses were made in GraphPad Prism 10.2.2 (GraphPad Software, Boston, USA). Joinpoint v. 5.2.0 (National Cancer Institute, USA) was used to analyze incidence trends and reported with annual percentage change (APC) with CI. A *P* <0.05 was considered statistically significant.

### Ethics

This study was granted ethical approval from the Swedish Ethical Review Authority (reference number 2023-00921-01). The need for informed consent was waived by the ethical review authority due to the retrospective study design.

## Results

### Baseline characteristics

During the study period, 202 patients experienced a total of 212 episodes of BSIs caused by *M. morganii*. One *M. morganii* BSI episode was excluded due to the blood being sampled postmortem, resulting in a total of 211 episodes in 201 patients. The median age of the included patients was 77 years (range 38-99), and 55 (27%) patients were women. Most cases (34%, *n* = 72) were seen in individuals aged 71-80 years (Figure S1). The median CCI score was 6 (range 0-14) and 51 patients (25%) were considered immunosuppressed. In total, three patients (1%) were previous drug users and none had ongoing intravenous drug abuse (Table 1). Of the 211 episodes, 137 (65%) were health care–related acquisitions of the infection, 46 (22%) were community-acquired, and 28 (13%) were nosocomial infections. During the study period, eight patients had two episodes and one patient had three episodes of *M. morganii* BSI.

**Table 1:** Baseline characteristics of included patients.

Baseline	*n*
Age, mean	76.2 (38-99)
Gender, male	145 (201)
Charlson comorbidity index, mean	6.2 (0-14)
Myocardial infarction	23 (11%)
Congestive heart failure	64 (32%)
Peptic ulcer disease	19 (9%)
Peripheral vascular disease	51 (25%)
Cerebrovascular incident	40 (20%)
Dementia	18 (9%)
Chronic obstructive pulmonary disease	15 (7%)
Connective tissue disease	6 (3%)
Hemiplegia	12 (6%)
Chronic kidney disease	27 (13%)
Leukemia	6 (3%)
Lymphoma	7 (3%)
AIDS	0 (0%)
Cancer	
-solid tumor	21 (10%)
-metastasized	26 (13%)
Liver disease	
-mild	8 (4%)
-moderate/severe	1 (0%)
Diabetes	
-uncomplicated	23 (11%)
-end-organ damage	47 (23%)
Intravenous abuse	
-previous	3 (1%)
-current	0 (0%)

Table 1 showing the baseline data of the 201 patients included in the study.

### M morganii BSI incidence and trend

**Figure 1:**
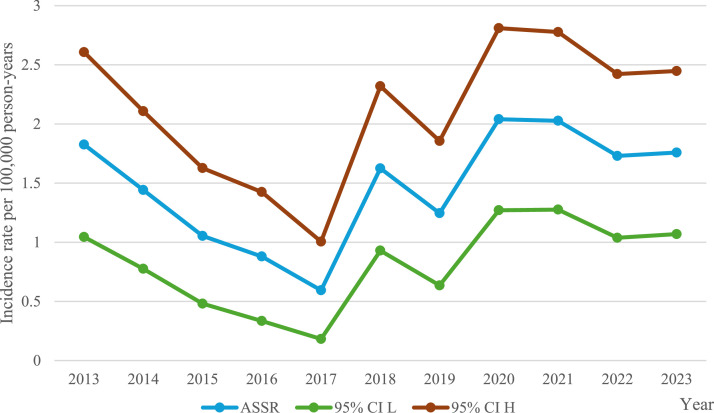
Incidence of *M. morganii* during the study period ASSR, age- and sex-standardized incidence rate; 95% CI L, confidence interval low; 95% CI H, confidence interval high.

The mean sex- and age-standardized incidence rate during the study period was 1.47 episodes per 100,000 person-years (Table S3). Between 2013 and 2016, there was a decrease in the incidence rate of *M. morganii* BSI; however, the decrease was not statistically different from zero (APC −22.25, 95% CI −46.6 to 0.87, *P* = 0.064). From 2017 to 2023, there was a statistically significant increase in episodes (APC 14.68, 95% CI 5.39 to 47.60, *P* = 0.014), reaching an incidence rate close to the baseline incidence of 2013 (Figure 1).

### Polymicrobial cultures and antimicrobial resistance rates

Of the 211 episodes, polymicrobial growth was seen in 85 (40%) episodes. Two bacteria were found in 43 (51%) episodes, three bacteria were seen in 24 (28%) episodes, and four bacteria were found in 15 (18%) episodes. Five and six bacteria were seen in two (2%) and one (1%) episode, respectively. Other than *M. morganii, Escherichia coli* was the most common bacteria found in 28 (33%) of the polymicrobial blood cultures, *Enterococcus faecalis* appeared in 20 (24%) of the cultures, and *Streptococcus anginosus* appeared in eight (10%) of the polymicrobial cultures. The polymicrobial cultures consisted of a total of 50 different bacteria (Table S2).

The rates of resistance toward cefotaxim was 8%, and no meropenem-, imipenem-, or piperacillin-tazobactam–resistant strains were found (Table S4).

### Intensive care and all-cause mortality

Patients were hospitalized for 206 (98%) of the 211 episodes, and the median number of days admitted was eight (range 0-106). In 14 episodes (7%), patients were admitted to the intensive care unit, with a median number of days of 3.5 (range 0-16). A total of 12 (6%) patients developed septic shock. The 30-, 90-, 180-, and 365-day all-cause mortality rates were 13% (*n* = 26), 19% (*n* = 39), 26% (*n* = 52), and 27% (*n* = 55), respectively.

### Bloodstream infection foci

The most common origin of the *M. morganii* BSIs was the urinary tract (50%, *n* = 105). Less common infection foci were primary BSI (24%, *n* = 50), skin-related infections (14%, *n* = 30), intra-abdominal source of infection (5%, *n* = 11), pneumonia (2%, *n* = 4), and osteomyelitis (1%, *n* = 1). A urinary tract-focused BSI was associated with reduced risk of 90-day mortality (*P* = 0.0029), whereas a primary BSI was associated with 90-day mortality (*P* = 0.0112) (Table 2).

**Table 2:** Bloodstream infection foci and association with 90-day mortality.

Infection focus				
	*n* = 211	90-day mortality *n* = 39	Alive *n* = 172	*P*-value
Urinary tract	105 (50%)	11 (11%)	94 (55%)	**0.0029**
Primary bloodstream infection	53 (25%)	16 (41%)	37 (22%)	**0.0112**
Skin	36 (17%)	8 (21%)	28 (16%)	0.5257
Intraabdominal	12 (6%)	3 (8%)	9 (5%)	0.5493
Pneumonia	4 (2%)	1 (3%)	3 (2%)	0.7346
Bone/joint	1 (0%)	0	1 (1%)	
Endocarditis	0			
Central nervous system infection	0			

### Risk factors for 90-day mortality

The unadjusted analyses found associations between immunosuppression (*P* = 0.0006) and higher CCI (*P* = 0.0003) with all-cause mortality within 90 days after *M. morganii* BSI. Patients who died within 90 days had significantly higher NEWS2 score (*P* = 0.01), higher respiratory frequency (*P* = 0.02), lower temperature (*P* = 0.04), higher Reaction Level Scale score (*P* = 0.03), and lower systolic blood pressure (*P* = 0.0005) at admission than survivors. Plasma lactate was significantly higher in the 90-days mortality group (*P* = 0.0005). Compared with survivors, significantly fewer patients had urinary tract symptoms (*P* = 0.0086) and simultaneous growth of *M. morganii* in urine culture (*P* = 0.0092) in the 90-day mortality group.

There was no difference between the groups regarding polymicrobial growth (Table 3).

**Table 3:** Univariate analysis of variables associated with 90-day mortality.

	90-day mortality, n = 39 (%)	Alive, n = 172	Total, n = 211	*P*-value
Gender, male	31 (79)	123 (72)	154 (73)	0.3355
Age, mean	77	76		0.5835
Charlson comorbidity index, mean	7.5	5.9		**0.0003**
Myocardial infarction	4 (10)	21 (12)	25 (12)	0.7333
Congestive heart failure	14 (36)	54 (31)	68 (32)	0.5870
Diabetes	13 (33)	62 (36)	75 (36)	0.7309
Dementia	3 (8)	16 (9)	19 (9)	0.7437
Connective tissue disease	0 (0)	7 (4)	7 (3)	0.1987
Chronic obstructive pulmonary disease	1 (3)	17 (10)	18 (9)	0.1375
Liver disease	3 (8)	8 (5)	11 (5)	0.4459
Chronic kidney disease	8 (2)	19 (11)	27 (13)	0.1135
Peripheral vascular disease	12 (31)	43 (25)	55 (26)	0.4587
Cerebrovascular incident	8 (2)	36 (21)	44 (21)	0.9404
Tumor	13 (33)	35 (20)	48 (23)	0.0842
Peptic ulcer disease	3 (8)	17 (10)	20 (9)	0.6659
Leukemia	3 (8)	5 (3)	8 (4)	0.1604
Lymphoma	2 (5)	5 (3)	7 (3)	0.4889
Hemiplegia	4 (10)	10 (6)	14 (7)	0.3193
Immunosuppression	19 (49)	37 (22)	56 (27)	**0.0006**
National early warning score 2, median	6	4		**0.0113**
Respiratory rate, median	22	20		**0.0211**
Temperature, mean	37.7	38.2		**0.0415**
Heart rate, mean	102.3	98.35		0.3197
Reaction level scale^a^ = 1	32 (82)	159 (93)	191 (91)	**0.0318**
Saturation, median	96	96		0.9389
Systolic blood pressure, mean	109.5	126.9		**0.0005**
Duration of symptoms, days median	1	1		0.3503
Abdominal pain	12 (31)	40 (23)	52 (25)	0.3354
Chills	11 (28)	66 (39)	77 (36)	0.2243
Urinary tract infection symptoms	4 (10)	53 (31)	57 (27)	**0.0086**
Plasma-C-reactive protein, median	110	121		0.5000
Plasma lactate, mean	4.1	2.7		**0.0005**
Plasma leucocytes, median	15.3	14.1		0.8417
Urine leucocytes present +(−)	3 (8)	49 (15)	52 (25)	0.4078
Urine leucocytes, mean	1.8	2.0		0.7812
Neutrophils, median	6.3	9.4		0.4788
Morganella morganii growth in urine	3 (8)	47 (27)	50 (24)	**0.0092**
Days with antibiotics, mean	9.256	14.32		**0.0002**
BSI with polymicrobial growth	18 (46)	66 (39)	84 (40)	0.3701

Chi-square for binary parameters, Mann–Whitney U-test for non-parametric parameters, or *t*-test for parametric parameters.

a Reference: Starmark JE, Stålhammar D, Holmgren E. The Reaction Level Scale (RLS85). Manual and guidelines. Acta Neurochir (Wien). 1988;91(1-2):12-20.

Empirical treatment with cefotaxime was associated with a reduced risk of 30- (*P* = 0.04) and 90-day mortality (*P* = 0.001) compared with non-cefotaxime antimicrobial treatment. This association was also seen for empirical treatment with cefotaxime, and empirical treatment modified to cefotaxime after *M. morganii* blood culture results were available for 30- (*P* = 0.03) and 90-day mortality (*P* = 0.0019). Treatment with piperacillin/tazobactam or carbapenems was not associated with increased risk of 30-day mortality (*P* = 0.11) but was associated with increased risk of 90-day mortality (*P* = 0.0011).

A multivariate analysis including age, gender, CCI, and immunosuppression revealed that higher CCI (OR 1.25, 95% CI 1.07-1.47, *P =* 0.0059) and immunosuppression (OR 3.26, 95% CI 1.45-7.47, *P* = 0.0045) were independently associated with all-cause mortality (Table S5).

## Discussion

We aimed to comprehensively investigate the incidence rates and temporal trend, clinical features, and outcome for *M. morganii* BSIs in southern Sweden. We found that *M. morganii* BSI is an infection of the elderly, with no cases seen in neonates, children, or young adults. We also found that *M. morganii* BSI is most often acquired in individuals that are in close contact with health care because only 22% of *M. morganii* BSI were community-acquired. The temporal trend revealed an increasing incidence during recent years, only to reach the baseline level of the start of the study. The rate of polymicrobial growth was high, observed in 40% of the episodes.

We found a mean sex- and age-standardized incidence rate of 1.47 episodes per 100,000 person-years during the study period, which is comparable to the largest *M. morganii* BSI cohort to date from Queensland, Australia that found an annual incidence of 9.2 cases per million population [7]. Our incidence is higher than what has been reported from British Colombia, Canada that estimated *M. morganii* BSI to be 0.5 per 100,000 population [8]. Comparable to these studies, *M. morganii* BSI primarily affects the elderly with multiple comorbidities, and the incidence rate increased over time. Whether this is due to increased sampling or a true increase is unknown because we have seen increased sampling in our setting [2]. A true incidence increase could have numerous causes, including nosocomial/health care–related spread and the dissemination of virulent clones. Virulent and multidrug-resistant lineages of *M. morganii* obtained from hospitalized patients have been described, which could facilitate such spread [14].

Unlike many other severe infections, the incidence of *M. morganii* BSI steadily increased during the COVID-19-pandemic. This could be explained by the fact that most our episodes were health care–related or nosocomial, which is in line with previous studies [14,15]. Previous reports describe *M. morganii* primarily as an opportunistic bacterium. However, only 25% of the patients in our study were immunocompromised, highlighting that *M. morganii* can be an important pathogen causing BSIs also in individuals with intact immune system.

The most common focus of infection in our study was the urinary tract (50%), which in line with a study from Taiwan that concluded that the *M. morganii* BSI originated from the urinary tract in 41.3% of the cases [16]. Interestingly, urinary origin only represented 13.7% in the Australian study [7]. Instead, primary BSI and soft tissue infection were the most common foci of infection, 45.8% and 18.5%, respectively. In our study, primary BSI and soft tissue infection constituted 24% and 14%, respectively.

The 30-day mortality rate of 13% in our study was very low compared with a Saudi Arabian study that found a 14-day mortality rate of 41% [17] and a study from Israel that reported an in-hospital mortality rate of 42% [18]. A possible explanation for the discordant results could be higher rates of antimicrobial resistance found in their studies. Our 30-day mortality rate of 13% is also lower than the Australian study that described a 21.2% 30-day mortality rate, with antimicrobial resistance rates more comparable to ours. This could be due to differences in health care systems and access to health car, and because patients managed in private institutions were not included in the Australian study.

Our analysis of risk factors for 90-day mortality revealed that higher CCI; immunosuppression; higher NEWS, respiratory rate, Reaction Level Scale score, temperature; higher plasma lactate; and lower blood pressure were associated with 90-day mortality in the unadjusted model. In addition, symptoms of urinary tract infection, *M. morganii* growth in urine, and a urinary tract focus was associated with a reduced risk of 90-day mortality, and a primary BSI was associated with higher risk of 90-day mortality. This is in line with previous studies that found, among other things, that a clinical syndrome other than urinary tract infection was a significant risk factor for mortality [7,18]. Comparable to this study, they found that more comorbidities with a higher CCI was associated with mortality because our adjusted model revealed an association between higher CCI and immunosuppression with 90-day all-cause mortality. Unlike a previous study, age was not a risk factor for 90-day all-cause mortality; however, this could be explained by the fact that we chose to model age linearly [17].

The rates of resistance toward the clinically important antimicrobials cefotaxime, meropenem, and piperacillin/tazobactam were very low in *M. morganii* isolates identified in this study. *M. morganii* possess inducible chromosomal AmpC β-lactamases, particularly, hospital-acquired strains, and exposure to β-lactams, especially third-generation cephalosporins, may lead to hyperproduction of these AmpC β-lactamases, with the development of resistance during antibiotic treatment [19]. We found no evidence of a clinically worse outcome in patients who received cefotaxime empirically or where treatment was altered to cefotaxime once the blood culture result of *M. morganii* was known. However, these results, as well as the association between empirical treatment with piperacillin/tazobactam or carbapenems with a higher 90-day mortality rate, could be explained by indication bias. For instance, carbapenems is reserved for patients who present with deranged vital signs, indicative of sepsis or septic shock. Few previous studies have reported septic shock as outcome; however, but we found a rate of septic shock (6%) that was very low compared with the 18.2% reported from Israel [18]. We found no *M. morganii* BSIs in children or neonates, which has previously been reported [20].

The strengths of our study include the population-based methodology, and the 11-year study period. We included all *M. morganii* BSIs and were able to access almost all medical records. The limitations of our study are inherent in the retrospective study design, with risks of selection bias and bias due to missing data. We only selected a few variables in the multivariate analyses that were regarded as clinically relevant.

Given that the proportion of elderly is expected to increase in the coming years [21], the incidence of *M. morganii* BSI is expected to rise and will likely continue to strain individuals and health care in the future. We need to anticipate that virulent, antimicrobial-resistant strains could constitute a challenge, particularly, for elderly individuals with comorbidities.

## Conclusion

*M. morganii* BSI is an infection of the elderly that is most often acquired in individuals with comorbidities that are in close contact with health care. A urinary tract focus was associated with a reduced risk of 90-day mortality, and higher CCI and immunosuppression were associated with 90-day all-cause mortality in a multivariate analysis. In our setting, the incidence has been increasing during recent years, and health care providers need to be vigilant of *M. morganii* BSI given that the proportion of individuals aged 60 years or older are expected to double in the coming years.

## Declarations of competing interest

The authors have no competing interests to declare.

## Appendix Supplementary materials

Image, application 1
