# Supplementary material for: Morganella morganii bloodstream infection affects the elderly in close contact with health care
Source: IJID Reg. 2024 Oct 24;13:100480. doi: 10.1016/j.ijregi.2024.100480 (PMC11582464; doi:10.1016/j.ijregi.2024.100480)
Supplement: Supplementary file 1 [file mmc1.docx]

**Supplementary material**

**Figure S1.** Age intervals of patients included

**Table S1.** Predetermined study protocol for medical chart review

| **Variable:** | **Definition:** |
| --- | --- |
|  |  |
| Journal available | *Yes/no* |
| Age |  |
| Sex | *man/woman* |
| Polymicrobial cultures | *yes/no (more than one pathogen in blood culture)* |
| If polymicrobial, what other bacteria than *M. morganii* was present | *Free text* |
| Previous blood culture with *M. morganii* | *Yes/no* |
| Urine culture taken | *Yes/no* |
| Urine culture obtained before antibiotics administered | *Yes/no* |
| Urine culture positive for *M. morganii* | *Yes/no* |
| Need for hospital care, connected to *M. morganii* infection | *Yes/no* |
| Date for start of hospital stay | *Date* |
| Date for end of hospital stay | *Date* |
| Need for intensive care | *Yes/no* |
| Date for start of stay at intensive care unit | *Date* |
| Date for end of stay at intensive care unit | *Date* |
| Date of death |  |
| Assessed cause of death | *Free text/missing* |
| Palliation (no antibiotics given) | *Yes/no* |
| Cause of infection | *1 = Community-acquired (patient came from home), 2 = Nosocomial (blood culture taken >48 hours after start of stay at hospital), 3 = Healthcare-associated (patients receiving intravenous therapy at home, receiving wound care, receiving specialised nurse-care, or had self-administered intravenous medication, all within 30 days of the infection. The patients only receiving oxygen at home were excluded from this category. Patient present at a hospital, haemodialysis clinic, or receiving intravenous chemotherapy, all within 30 days of the infection. Permitted to care in an emergency hospital at least two days within 90 days of the infection. Lived in a nursing home or long-term care facility.* |
| Charlson score |  |
| Age | *< 50 years 0 p, 50-59 years 1 p, 60-69 years 2 p, 70-79 years 3 p, ≥ 80 years 4 p* |
| Myocardial infarction | *yes=1 p no=0p* |
| Congestive heart failure | *yes=1 p, no=0p* |
| Peripheral vascular disease | *yes (Intermittent claudication, previous bypass surgery, gangrene/acute ischemia, aortic aneurysm ≥ 6 cm) =1, no=0p.* |
| Cerebrovascular accident | *yes (previous stroke or transitory ischaemic attack) =1 p no=0p.* |
| Dementia | *yes=1 p, no=0p* |
| Chronic obstrucitve pulmonary disease | *yes=1 p, no=0p* |
| Connective tissue disease | *yes=1 p, no=0p* |
| Peptic ulcer disease | *yes (Previous proton pump-inhibitor/peptic ulcer) =1 p, no=0p* |
| Liver disease | *mild (chronic hepatitis or cirrhosis without portal hypertension) =1 p, moderate to severe (cirrhosis and portal hypertension with or without variceal bleeding) =3 p* |
| Diabetes | *none or diet controlled =0p, uncomplicated =1 p, end-organ damage =2 p* |
| Hemiplegia | *yes=2 p, no=0 p* |
| Moderate to severe chronic kidney disease | *yes (dialysis, kidney-transplanted, uraemia or creatinine > 270 µmol/L) =2 p, no=0 p* |
| Solid tumour | *no, =0 p, localized=2 p, metastasized=6 p* |
| Leukemia | *yes=2 p, no=0p* |
| Lymphoma | *yes=2 p, no=0 p* |
| AIDS (acquired immunodeficiency syndrome) | *yes= 6 p, no=0 p* |
| Total score |  |
| Immunosuppression | *Categories: 0 - None. 1- organ transplanted with ongoing immunosuppressing medication. 2- corticosteroid treatment >15 mg prednisolone or equivalent. 3- previous stem cell transplantation. 4- primary immune defect. 5- ongoing or recently terminated cancer-treatment such as chemotherapy, dialysis or severe chronic kidney disease. 6 - ongoing treatment for autoimmune disease* |
| Intravenous drug-abuse | *0=none, 1=active, 2=previous* |
| Antibiotics prior to blood culture | *Yes/no* |
| Empirical antibiotics | *Free text (type of antibiotic)* |
| Date for start of empiric treatment | *Date* |
| Date for end of empiric treatment | *Date* |
| Change to different antibiotics after blood culture result | *Yes/no* |
| If yes | *1 = piperacillin/tazobactam, 2 = imipinem, 3 meropenem, 4 other in free text* |
| Peroral step-down antibiotics | *Type of antibiotic* |
| Total days of antibiotic treatment |  |
| Duration of symptoms | *Number of days from start of symptoms to visit at emergency clinic* |
| Symptoms of urinary tract infection | *Yes/no* |
| Positive nitrate on urine dipstick | *Detection limit =13-22 µmol/L* |
| Amount of + on urine dipstick for leukocytes | *15-70 Leu/µL =1+, 70-125 Leu/µL =2+, 125-500 Leu/µL =3+, > 500 Leu/µL =4+* |
| Abdominal pain | *Yes/no* |
| Fever | *Temperature >38 ¤C, within 48 h or in anamnesis* |
| Chills | *Yes/no* |
| Systolic blood pressure at emergency clinic | *mm/Hg* |
| Pulse | *Beats per minute* |
| Respiratory frequency | *breaths per minute* |
| Score on reaction level scale | *1-8 p* |
| Saturation | *0-100%* |
| Temperature | *degrees Celsius* |
| Total score on NEWS2 at emergency clinic |  |
| Radiology done for abdominal/urinary organs | *Yes/no* |
| Signs of pyelonephritis | *Yes/no (assessed by either physician at the time of care or by the study researchers from journal information)* |
| Septic shock | *Yes/no (according to SEPSIS-3 criteria)* |
| Leucocyte partial concentration | *10^9 /L (Within 48 hours of blood culture taken)* |
| Neutrophils | *10^9 /L (Within 48 hours of blood culture taken)* |
| C-reactive protein (CRP) | *mg/L (Within 48 hours of blood culture taken)* |
| Lactate | *mmol/L (Within 48 hours of blood culture taken)* |
| Sick leave after termination of stay | *Yes/no* |
| Domestic help (new) after termination of stay |  |
| ICD-code or treating physicians’ diagnosis at end of stay |  |
| Focus of infection | *1 = No focus determined, 2 = Skin, 3 = bone/joint, 4 = pneumonia, 5 = endocarditis/vascular-graft, 6 = Central nervous system, 7 = intraabdominally, 8 = urinary tract* |

**Table S2**. Bacteria in polymicrobial cultures

| **Bacterium:** | ***n*** |
| --- | --- |
| *E. coli* | 28 (33%) |
| *Enterococcus faecalis* | 20 (24%) |
| *Streptococcus anginosus* | 8 (10%) |
| *Klebsiella oxytoca* | 7 (8%) |
| *Bacteroides fragilis* | 6 (7%) |
| *Proteus mirabilis* | 6 (7%) |
| *Coagulase-negative staphylococci* | 5 (6%) |
| *Staphylococcus aureus* | 5 (6%) |
| *Proteus vulgaris* | 4 (5%) |
| *Pseudomonas aeruginosa* | 4 (5%) |
| *Aerococcus sanguinicola* | 3 (4%) |
| *Aerococcus urinae* | 3 (4%) |
| *Citrobacter freundii* | 3 (4%) |
| *Klebsiella pneumoniae* | 3 (4%) |
| *Providencia rettgeri* | 3 (4%) |
| *Serratia mercescens* | 3 (4%) |
| *Actinotignum species* | 2 (2%) |
| *Bilophila wadsworthia* | 2 (2%) |
| *Clostridium ramosum* | 2 (2%) |
| *Enterococcus faecium* | 2 (2%) |
| *Enterococcus raffinosus* | 2 (2%) |
| *Bacillus cereus* | 1 (1%) |
| *Bacteroides species* | 1 (1%) |
| *Bacteroides thetaiotaomicron* | 1 (1%) |
| *Clostridium difficile* | 1 (1%) |
| *Enterobacter cloacae* | 1 (1%) |
| *Enterococcus avium* | 1 (1%) |
| *Enterococcus gallinarum* | 1 (1%) |
| *Enterococcus hirae* | 1 (1%) |
| *Fusobacterium nucleatum* | 1 (1%) |
| *Fusobacterium species* | 1 (1%) |
| *Group A streptococci* | 1 (1%) |
| *Group B streptococci* | 1 (1%) |
| *Gemella morbillorum* | 1 (1%) |
| *Klebsiella variicola* | 1 (1%) |
| *Lactobacillus* | 1 (1%) |
| *Parvimonas micra* | 1 (1%) |
| *Proteus species* | 1 (1%) |
| *Pseudomonas aeruginosa* | 1 (1%) |
| *Ruminococcus gnavus* | 1 (1%) |
| *Staphylococcus anginosus* | 1 (1%) |
| *Staphylococcus hominis* | 1 (1%) |
| *Staphylococcus Epidermidis* | 1 (1%) |
| *Streptococcus bovis* | 1 (1%) |
| *Streptococcus constellatus* | 1 (1%) |
| *Streptococcus gordonii* | 1 (1%) |
| *Streptococcus mitis* | 1 (1%) |
| *Trueperella bernardiae* | 1 (1%) |
| *Veillonella species* | 1 (1%) |

**Table S3. Crude and sex- and age-standardized incidence rates**

| **Year** | **Crude rate** | **ASSR** | **95%CI L** | **95% CI H** |
| --- | --- | --- | --- | --- |
| 2013 | 1.65 | 1.83 | 1.04 | 2.61 |
| 2014 | 1.40 | 1.44 | 0.78 | 2.11 |
| 2015 | 1.00 | 1.05 | 0.48 | 1.63 |
| 2016 | 0.75 | 0.88 | 0.33 | 1.42 |
| 2017 | 0.59 | 0.59 | 0.18 | 1.01 |
| 2018 | 1.54 | 1.62 | 0.93 | 2.32 |
| 2019 | 1.16 | 1.25 | 0.64 | 1.86 |
| 2020 | 1.94 | 2.04 | 1.27 | 2.81 |
| 2021 | 2.00 | 2.03 | 1.28 | 2.78 |
| 2022 | 1.70 | 1.73 | 1.04 | 2.42 |
| 2023 | 1.76 | 1.76 | 1.07 | 2.45 |
| Mean | 1.41 | 1.47 | 0.82 | 2.13 |

ASSR = age- and sex-standardized rate. CI L = confidence interval low, CI H = confidence interval high.

**Table S4.** Antimicrobial susceptibility for *M. morganii* BSI.

| **Antimicrobial** |  |  |  |
| --- | --- | --- | --- |
|  | Resistant *n* | Isolates tested n | Resistance rate % |
| Amikacin | 0 | 53 | 0 |
| Amoxicillin | 1 | 1 | 100 |
| Amoxicillin/Clavulanic acid | 25 | 26 | 96 |
| Ampicillin | 20 | 20 | 100 |
| Benzylpenicillin | 1 | 1 | 100 |
| Cefadroxil | 145 | 147 | 99 |
| Cephalexin | 11 | 12 | 92 |
| Cefotaxime | 17 | 210 | 8 |
| Ceftazidime | 16 | 211 | 8 |
| Ceftriaxone | 0 | 1 | 0 |
| Ciprofloxacin | 15 | 210 | 7 |
| Colistin | 2 | 2 | 100 |
| Ertapenem | 0 | 53 | 0 |
| Gentamicin | 9 | 209 | 4 |
| Imipenem | 0 | 201 | 0 |
| Meropenem | 0 | 210 | 0 |
| Piperacillin/tazobactam | 0 | 210 | 0 |
| Tigecycline | 20 | 20 | 100 |
| Tobramycin | 1 | 210 | 0 |
| Trimetoprim | 5 | 53 | 9 |
| Trimethoprim/sulfamethoxazole | 26 | 205 | 13 |

**Table S5.** Multivariate logistic regression of selected variables associated with 90-day mortality

| **Variable** | **OR (95% CI)** | ***p*-value** |
| --- | --- | --- |
| Age | 1.02 (0.98-1.06) | 0.2946 |
| Gender | 0.61 (0.23-1.44) | 0.2804 |
| Charlson comorbidity index | 1.25 (1.07-1.47) | **0.0059** |
| Immunosuppression | 3.26 (1.45-7.47) | **0.0045** |

OR = odds ratio, CI = confidence interval.
